# One-step synthesis of zwitterionic graphene oxide nanohybrid: Application to polysulfone tight ultrafiltration hollow fiber membrane

**DOI:** 10.1038/s41598-020-63356-2

**Published:** 2020-04-23

**Authors:** G. P. Syed Ibrahim, Arun M. Isloor, A. F. Ismail, Ramin Farnood

**Affiliations:** 1 Membrane and Separation Technology Laboratory, Chemistry Department, National Institute of Technology, Karnataka Surathkal, Mangalore, 575 025 India; 2 Apahatech Solutions LLP, Science & Technology Entrepreneurs Park, National Institute of Technology Karnataka, Surathkal, Mangalore, 575 025 India; 3 Advanced Membrane Technology Research Center (AMTEC), Universiti Teknologi Malaysia, 81310 Skudai, Johor Bahru Malaysia; 4 Department of Chemical Engineering and Applied Chemistry, University of Toronto, Toronto, ON M5S 3ES, Canada

**Keywords:** Environmental sciences, Chemistry, Materials science, Nanoscience and technology

## Abstract

In this paper, novel zwitterionic graphene oxide (GO) nanohybrid was synthesized using monomers [2-(Methacryloyloxy)ethyl]dimethyl-(3-sulfopropyl)ammonium hydroxide (SBMA) and N,N′-methylenebis(acrylamide) (MBAAm) (GO@poly(SBMA-co-MBAAm), and incorporated into polysulfone (PSF) hollow fiber membrane for the effectual rejection of dye from the wastewater. The synthesized nanohybrid was characterized using FT-IR, PXRD, TGA, EDX, TEM and zeta potential analysis. The occurrence of nanohybrid on the membrane matrix and the elemental composition were analyzed by XPS. The as-prepared tight ultrafiltration hollow fiber membrane exhibited high rejection of reactive black 5 (RB-5, 99%) and reactive orange 16 (RO-16, 74%) at a dye concentration of 10 ppm and pure water flux (PWF) of 49.6 L/m^2^h. Fabricated nanocomposite membranes were also studied for their efficacy in the removal of both monovalent (NaCl) and divalent salts (Na_2_SO_4_). The results revealed that the membrane possesses complete permeation to NaCl with less rejection of Na_2_SO_4_ (<5%). In addition, the nanocomposite membrane revealed outstanding antifouling performance with the flux recovery ratio (FRR) of 73% towards bovine serum albumin (BSA). Therefore, the in-house prepared novel nanocomposite membrane is a good candidate for the effective decolorization of wastewater containing dye.

## Introduction

In recent times, wastewater generated from the textile industries becoming detrimental environmentally demanding effluent. It is mainly composed of a large amount of organic dyes and inorganic salts, properly ~5.6 wt% of Na_2_SO_4_ and ~6 wt% of NaCl^1,2^. Among the organic dyes used, reactive dyes have gained significant attention owing to the low tendency of fixing on the fibers. Accordingly, a considerable amount of inorganic salts should be used to enhance the binding capability, which led to the existence of a large number of dyes and salts in the wastewater stream^3^. The discharge of untreated wastewater effluents into the environment, not only affect human beings but also restrict the permeation of light which will affect the aquatic flora and fauna to a greater extent^4–8^. Further, the discharged dye molecules are highly vulnerable to hydrolysis or oxidation to degrade into different toxic substances. Reactive dyes are capable of causing skin diseases like contact dermatitis and respiratory diseases like asthma^9^. In the matter of sustainability, dyes and inorganic salts should be separated from the wastewater and recycled rather than dye removal or water purification by reverse osmosis (RO). Additionally, the recycled dye can be used in the dyeing process and salts can work well as draw solution in forward osmosis (FO)^10–12^.

Membrane separation is one of the efficient and popular techniques for the purification of wastewater^13–17^. In the 1980s, the nanofiltration (NF) membrane was introduced and has become an attractive technology due to the improved selectivity, high permeation, low operating pressure and less maintenance cost^18–21^. NF membrane has a pore size of ~0.5–2.0 nm and molecular weight cut-off (MWCO) between 200 and 1000 Da, which renders the opportunity to stand superior in wastewater purification^22^. The mechanism of NF membrane separation is based on size exclusion and electrostatic repulsion (Donnan effect). However, NF membrane performances are severely affected by concentration polarization and membrane fouling^23^. Essentially, in textile wastewater treatment, the flux decline is triggered by the cake layer formation and pore blockage^11,24^. According to Koyuncu, membrane fouling can be reduced by increasing the cross-flow velocity^25^. In addition, the hydrophobic interaction and electrostatic attraction will also contribute to the reduced flux via the accumulation of dye molecules in the membrane pore structure^26^. Consequently, it increases the frequency of chemical cleaning, which reduces membrane life. Besides, the high concentration of inorganic salts reduces the permeate flux due to the increase in osmotic pressure^27^. Therefore, advanced NF membranes having dye removal capacity with low salt rejection and high flux are highly anticipated in the field of textile wastewater treatment.

The usage of tight ultrafiltration (T-UF) can be recognized as an operative approach for the bifurcation of dye/salt mixture^28,29^. Lin *et al*. demonstrated the fractionation of dye/Na_2_SO_4_ using the UH004 T-UF membrane (MWCO 4700 Da) from textile wastewater. The results revealed thatmembrane could reject >98.9% of both reactive blue 2 and direct dyes along with the high permeation of Na_2_SO_4_^28^. Liu *et al*. applied positively charged T-UF membrane with MWCO of 12700 Da for separation of dye, which allowed 99.9% rejection of Congo red with dye solution permeation of 84 L/m^2^h^30^. Hydrophilic polyaryletherketone (PAEK)-COOH T-UF membrane was reported by the Mao group. The prepared membrane exhibited rejection of 99.8% Congo red (100 ppm) with the complete permeation of NaCl and <10% rejection to Na_2_SO_4_^29^. Hence, the T-UF membrane having both the high flux of water and inorganic salts with high rejection of dye molecules is highly desirable since the textile effluents increase day by day.

The carbon-based nanomaterials have been demonstrated many interesting properties such as adsorption of organic dyes and heavy metals, desalination and antimicrobial activity^31–34^. Graphene oxide (GO) consists of reactive functional groups such as -OH, epoxy, and -COOH has designated as an effective substitute for constructing the nanocomposite membranes owing to its characteristic 2D structure, high chemical stability, strong hydrophilicity, and high surface area^35^. GO has been incorporated into the membrane matrix for constructing nanocomposite membranes with enhanced flux and antifouling properties^36–38^. Nevertheless, graphene derivatives are susceptible to form aggregation in dope solution, as a result, it forms a random dispersion. Furthermore, the compatibility between the membrane matrix and GO is poor, which may enhance the defects in the matrix. Consequently, there will be a compromise in the selectivity and mechanical strength of the nanocomposite membrane^1,39^. For that reason, surface modification of GO for the betterment of improved performance and compatibility is mandatory. Zwitterionic materials bearing both anionic and cationic groups have been developed as favorable antifouling and superhydrophilic materials. It establishes a strong hydration layer on the membrane surface, which is essential for reducing the adsorption of foulants capability and enhancement of flux^40^.

The nanocomposite membrane will have improved compatibility, as the hydrophobic chains in the zwitterionic polymers are miscible with the polymer matrix. Zhu *et al*. described the preparation of zwitterionic GO via reverse atom transfer radical polymerization (RATRP), which is overall in three steps^1^. Zhao *et al*. prepared zwitterionic GO via free radical polymerization at 60 °C under a nitrogen atmosphere. The prolonged reaction time (40 h) and inert atmosphere still increase the operational cost^41^. He *et al*. stated the functionalization of GO in two steps. In the first step, GO was vinylated by 3-(methacryloxy) propyltrimethoxysilane (MPS) followed by reaction with some of the monomers via surface-initiated precipitation polymerization^42^.

Distillation precipitation polymerization (DPP) is one of the developing methods for the preparation of uniform-sized micro and nanoparticles. It was first reported by Bai *et al*. for the preparation of poly(divinylbenzene) microspheres^43^. It is described that the size of particles can be altered by changing the concentration of the monomer and radical initiator. In addition, the increased degree of cross-linking also influences the size of the particles. DPP is i) Time-saving (<2 h) and facile method compared to the conventional polymerization process. ii) No stabilizer and pH adjustment are required. iii) As reaction proceeds at refluxing temperature, no need for the inert atmosphere. iv) Prepared particles show excellent colloidal stability in various solvents without adding any stabilizing agents. In the typical process, acetonitrile (ACN) was chosen as a reaction solvent, since the monomers and initiator are highly soluble and synthesized nanoparticles are insoluble. Xihao *et al*. described the preparation of functionalized Fe_3_O_4_ nanoparticles via DPP. The as-prepared hydrophilic nanoparticles demonstrated the improved binding capacity towards glycoproteins^44^. Guangwei *et al*. modified the MWCNTs with functional groups such as –COOH, –SO_3_H and PO_3_H_2_ by DPP. It was stated that the modified MWCNTs improved proton conductivity^45^.

Jianfeng *et al*. reported the first paper on the surface functionalization of multiwalled carbon nanotube (MWCNTs) with poly(acrylic acid) and poly(acrylamide) via *in situ* radical polymerization^46^. Later, the GO surface was functionalized with poly(acrylic acid) and poly(acrylamide) via covalent bonding, which was reported by Mingxin *et al*.^47^. In 2011, Kan *et al*. demonstrated the functionalization of GO with different polymer chains via free radical polymerization^48^. It was stated that after functionalization, the aggregation of GO was reduced to a greater extent. In this typical reaction, polymer chains are directly attached to the C=C bond of GO and the rest of the active functional groups were intact. Further, by combining the advantages of this method and DPP would provide a simple and cost-effective methodology for the functionalization of GO. In this typical polymerization process, the vinyl monomers are initiated by radical polymerization and the formed radicals are added to C=C bonds of GO via propagation. Consequently, there is a formation of many radicals on the GO surface, which undergoes further chain propagation and termination. Conversely, so far no experimental evidence has been reported to support the above-said mechanism^49^. Additionally, the functional groups such as –COOH and –OH of GO are intact. Thus, there was no compromise in the hydrophilicity of GO after functionalization.

Inspired by the above reports, GO@poly(SBMA-co-MBAAm) nanohybrid was synthesized via DPP using GO as the backbone, MBAAm as a cross-linking agent and SBMA as a zwitterionic monomer. This material was used as an additive to prepare tight ultrafiltration (T-UF) hollow fiber (HF) membranes, using polysulfone (PSF) as the membrane material. The presence of all the elements and membrane morphology were studied using XPS and SEM analyses. The effect of incorporated nanocomposite on T-UF HF membranes was examined based on the surface hydrophilicity, water uptake, MWCO, and pure water flux. The as-prepared membrane’s filtration performance was demonstrated for dye and salt separation using negatively charged reactive dyes as model pollutants in detail. Additionally, the dye rejection at different concentrations of dye and salt, different pH, various pressure and antifouling performances have also been studied. To the best of our understanding, this is the first such paper which describes the synthesis of novel GO@poly(SBMA-co-MBAAm) nanohybrid and used as an additive in PSF T-UF HF membrane.

## Materials and methods

### Materials

Azobisisobutyronitrile (AIBN), graphite powder, acetonitrile (ACN), N-methyl-2-pyrrolidone (NMP), polyvinylpyrrolidone (PVP) and MBAAm were obtained from Loba chemicals. Polysulfone pellets (PSF, Udel^®^ P-1700) was purchased from Solvay chemicals. SBMA, RB-5, RO-16 and bovine serum albumin (BSA) were procured from Sigma Aldrich and the molecular structure of reactive dyes is depicted in Fig. S1, Supporting Information. Sodium sulfate (Na_2_SO_4_), sulfuric acid (H_2_SO_4_), sodium chloride (NaCl), hydrogen peroxide (H_2_O_2_), potassium permanganate (KMnO_4_), phosphoric acid (H_3_PO_4_) acquired from Merck.

### Preparation of graphene oxide

Graphene oxide (GO) was prepared as stated in the literature^50^. In brief, 2 g of graphite was added to the mixture of 240 mL of Conc.H_2_SO_4_ and 27 mL of H_3_PO_4_ at 10 °C. 12 g of KMnO_4_ was added lot-wise under cooling. The reaction mass was cooled to RT and heated to 50 °C for 12 h. Furthermore, the reaction mass was cooled to RT and slowly poured into 130 mL of ice-cooled water containing 2 mL of 30% H_2_O_2_. The supernatant was decanted and the remaining solid was again washed with 130 mL of Conc. HCl (30%). The GO nanosheets were washed with water until the neutral pH and finally with 1 × 130 mL of ethanol and isolated by centrifugation.

### Synthesis of zwitterionic graphene oxide nanohybrid (GO@poly(SBMA-co-MBAAm))

**Figure 1:**
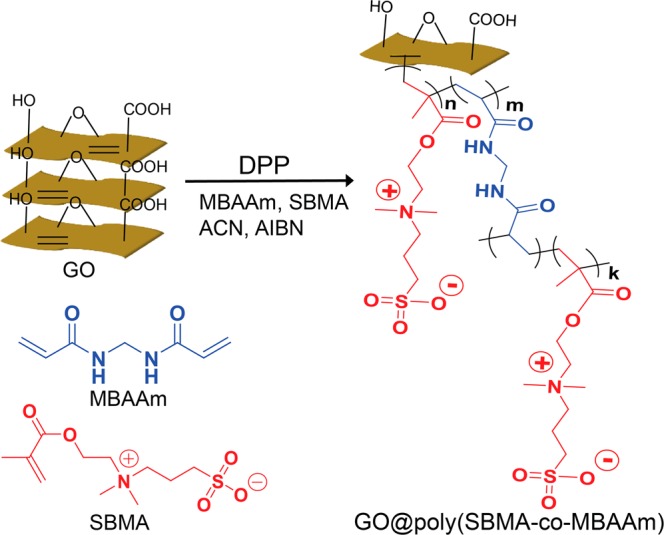
GO@poly(SBMA-co-MBAAm) nanohybrid synthesis.

The GO@poly(SBMA-co-MBAAm) nanohybrid was synthesized via a one-step DPP technique. Typically, 0.1 g of GO was dispersed in 100 mL of acetonitrile in a dried round bottom flask by using an ultrasonic cleaning bath (Anmanm industries USC-100). Added 0.2 g (0.71 mmol) of SBMA, 0.8 g (5.18 mmol) of MBAAm and 0.02 g (0.12 mmol) of AIBN and stirred for 15 min at room temperature. The reaction mass was purged with nitrogen gas for 20 min to eliminate the dissolved oxygen. Later the reaction was performed by attaching Dean-Stark receiver and heated in an oil bath to 75 °C for another 15 min. The oil bath temperature was gradually rose to 110 °C to continue the polymerization reaction at reflux. When 40 mL of acetonitrile has removed over 40 min through Dean-Stark receiver, the reaction mixture allowed to room temperature and agitated for an additional 1 h. The nanoparticles were isolated and washed with acetonitrile to remove the oligomer and unreacted monomer. The material was dried at 55 °C for 15 h (Yield = 0.9 g). The synthetic route of GO@poly(SBMA-co-MBAAm) nanohybrid is represented in Scheme 1 (as seen in Figure 1).

### Preparation of tight ultrafiltration hollow fiber membranes

PSF tight ultrafiltration (T-UF) hollow fiber (HF) membranes were prepared by the dry/wet phase inversion method. The PSF and PVP were dried at 60 °C for 24 h prior to dope solution preparation to eliminate the adsorbed water molecules. Details of the spinning parameter and dope solution composition are depicted in Tables S1 and S2. In the preparation of TM-2, 0.05 g of GO@poly(SBMA-co-MBAAm) nanohybrid dispersed in 79 g of NMP using a probe sonicator (QSONICA, 50% amplitude with on time of 1 second and off time of 10 seconds) for 10 min. Added 1 g of PVP and stirred for 10 min at room temperature. 20 g of PSF was added to the dope solution lot wise and stirred at 60 °C for 24 h to obtain the homogeneous solution. The dope solution was degassed for 6 h to remove any residual air bubbles. The as-prepared dope solution was used to spin the T-UF HF membrane. The schematic representation of the HF membrane spinning system is represented in our previous report^51^. The as-made HF membranes were dipped in deionized water for 24 h to complete the phase inversion followed by 20 wt% glycerol solution for another 24 h to circumvent any pore collapse. The membranes were air-dried for future usage. The membranes prepared with different GO@poly(SBMA-co-MBAAm) concentration of 0, 0.1, 0.25 and 0.5 wt% from now denoted to as TM-0, TM-1, TM-2 and TM-3.The details of the characterization of nanohybrid and membranes are provided in the Supporting Information.

## Results and discussion

### Characterization of GO@poly(SBMA-co-MBAAm) nanohybrid

**Figure 2:**
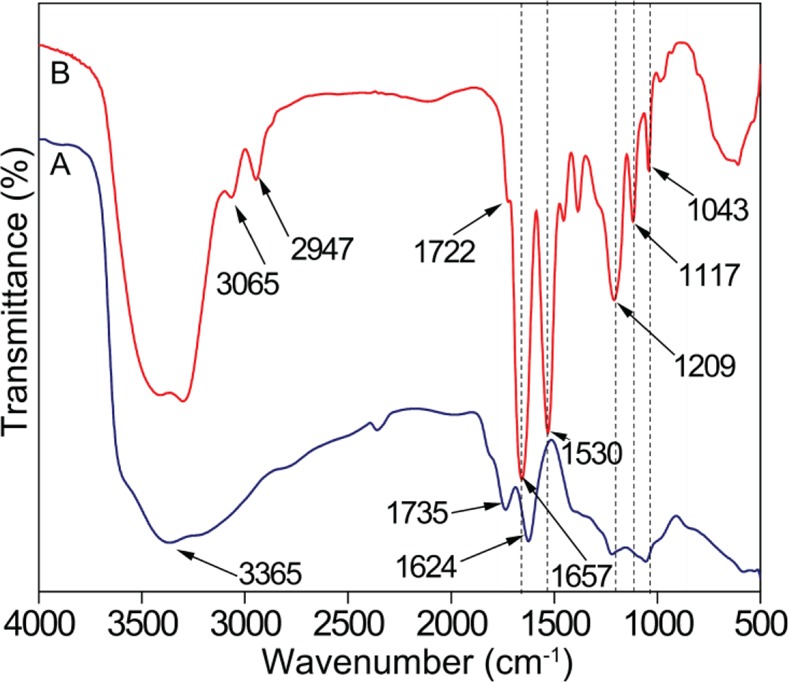
FT-IR spectra of (**A**) GO and (**B**) GO@poly(SBMA-co-MBAAm).

GO and GO@poly(SBMA-co-MBAAm) were examined by FT-IR and are presented in Figure 2. In GO (Figure 2A), the peak at 3365 cm^−1^ due to O-H stretching vibration and the C=O peak was observed at 1735 cm^−1^. The peaks at 1624, 1219 and 1056 cm^−1^ were owing to C=C, C-O, and C-O-C vibrations respectively^52^. ForGO@poly(SBMA-co-MBAAm), peaks at 3065 and 2947 were ascribed to C-H stretching vibrations. The ester group present in SBMA was observed at 1722cm^−1^ ^41^. The amide group C=O stretching and N-H bending vibrations were detected at 1657 and 1530 cm^−1^. More importantly, peaks at 1043 and 1117 cm^−1^ were assigned to the symmetric and asymmetric stretching vibrations of the sulfonate (SO_3_^−^) group. The peak at 1209 cm^−1^ was bestowed by C-N stretching vibration^53^. In that way, the modification of GO was confirmed by FT-IR. Furthermore, TM-0 and TM-2 HF membrane ATR-FTIR spectra were also recorded and presented in Figure S2, Supporting Information. As shown, the peaks at 1293 cm^−1^, 1242 cm^−1^ and 1150 cm^−1^ were attributed to the characteristic S=O asymmetric stretch, C-O-C stretch and S=O symmetric stretch of PSF. The peak at 1662 cm^−1^ in both TM-0 and TM-2 membrane due to amide C=O stretch of residual PVP. The new peak at 1734 cm^−1^ in TM-2 membrane was due to the C=O stretch (carboxylic acid group) of GO, which confirmed the presence of nanomaterial in the TM-2 matrix. In addition, there was no appreciable shift in stretching frequency was observed in addition to PSF characteristic peaks by adding GO@poly(SBMA-co-MBAAm), which indicates no interaction between GO@poly(SBMA-co-MBAAm) and PSF chain.

**Figure 3:**
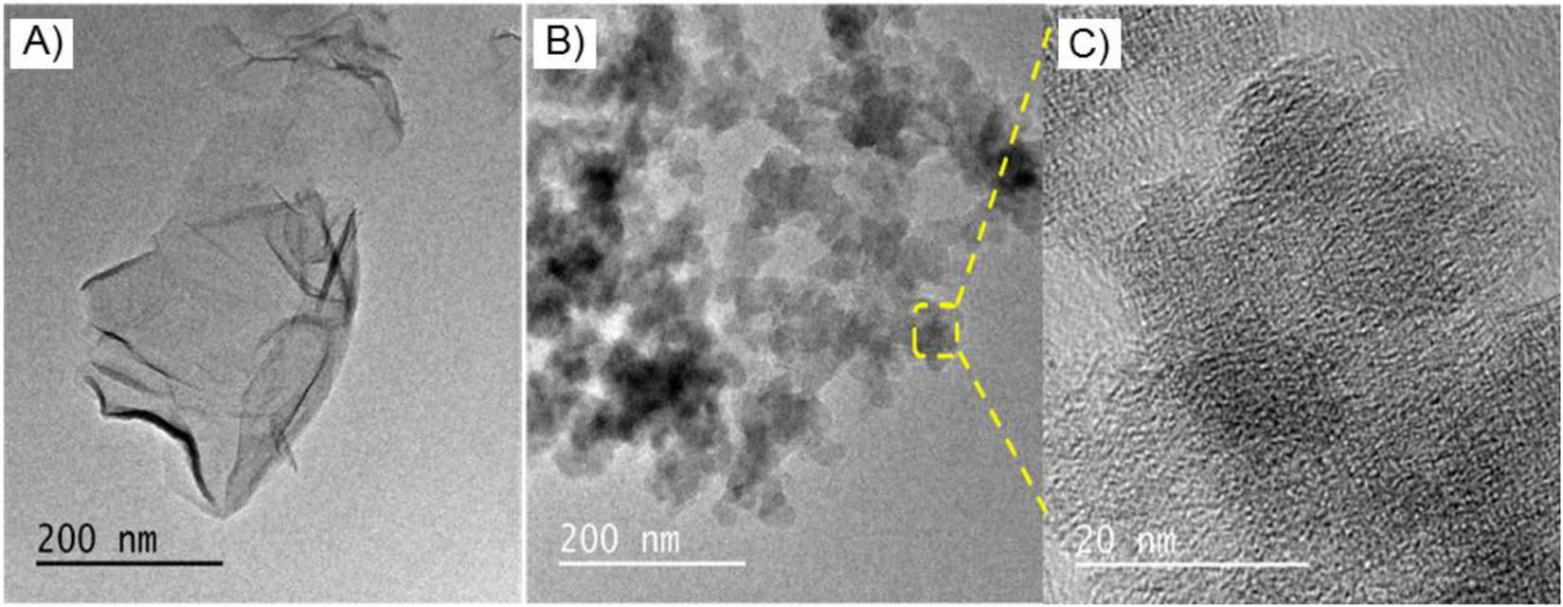
TEM images of (**A**) GO, (**B**,**C**) GO@poly(SBMA-co-MBAAm) in different magnifications.

The morphology of zwitterionic GO was observed in TEM and shown in Figure 3. In Figure 3A, GO exhibits a distinct crystal clear and distorted laminar structure. However, in Figure 3B, GO is uniformly covered by nanosize poly(SBMA-co-MBAAm) nanoparticles, which are homogeneously spread on the surface of GO. The surface of the GO was comparatively smooth and no wrinkles were observed. Conversely, the modified GO shown some of the wrinkles and folding under the same condition. In addition, many irregular dark dots are present on the modified GO surface, which makes the modified GO darker and rougher than unmodified GO. The magnified image of functionalized GO is shown in Figure 3C, which further confirmed that some of the poly(SBMA-co-MBAAm) nanoparticles are intercalated between the GO layers^54^. In summary, the morphological changes in TEM images confirmed the functionalization of GO.

**Figure 4:**
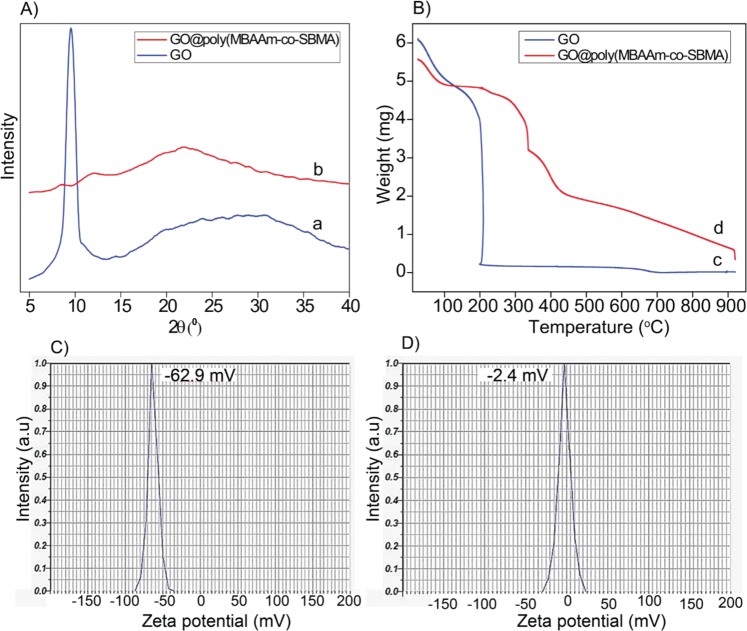
(**A**) PXRD of (a) GO, (b) GO@poly(SBMA-co-MBAAm), (**B**) TGA curve of (c) GO, (d) GO@poly(SBMA-co-MBAAm), (**C**,**D**) zeta potential of GO and GO@poly(SBMA-co-MBAAm).

Figure 4A represents the powder X-ray diffraction (PXRD) pattern of both GO and GO@poly(SBMA-co-MBAAm). GO has peak at 2θ = 9.8°, resultant to the [001] diffraction peak with an interlayer spacing of 9.0 Å^50,55,56^. However, in the case of GO@poly(SBMA-co-MBAAm), the [001] reflection of GO at 2θ = 9.8° was not detected as the regular stack of GO was demolished by the intercalation of nanosize poly(SBMA-co-MBAAm) and the increased disorder, which provides additional confirmation of intercalation of the poly(SBMA-co-MBAAm) at GO. The obtained result is well aligned with the literature^57–61^. Furthermore, the increased interlayer spacing and disorder of the GO@poly(SBMA-co-MBAAm) nanohybrid would have more amount of active sites than the GO.

TGA was used to understand the thermal properties of GO and GO@poly(SBMA-co-MBAAm) and presented in Figure 4B. In GO, the weight loss below 100 °C owing to adsorbed water. The major weight loss around 200 °C was due to CO, CO_2_ and steam release^62,63^ and slower weight loss between 450 and 900 °C was ascribed to the decomposition of more stable oxygen-containing functionalities^64^. In addition, the total weight loss of GO was ca. 65.1%. However, in the case of GO@poly(SBMA-co-MBAAm) the total weight loss was ca. 80.8%. The difference in weight loss validates the successful functionalization of GO. Therefore, the loading density of MBAAm and SBMA on GO was ca. 157 mg/g.

**Figure 5:**
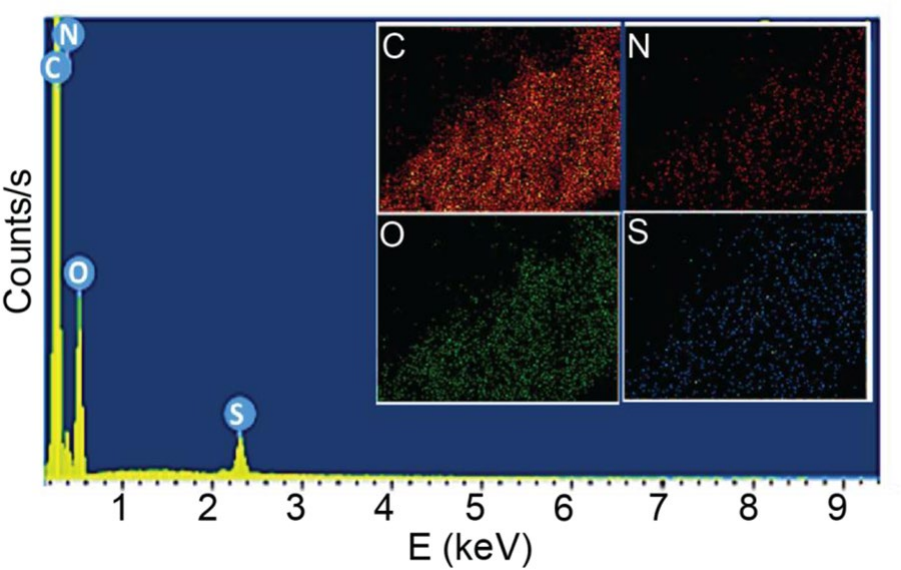
EDX and elemental mapping (inset) analyses of GO@poly(SBMA-co-MBAAm).

The surface charge of the nanohybrid was analyzed and depicted in Figure 4C and D. As far as the GO is concerned, it forms very stable aqueous colloids^65,66^. The prepared GO (Figure 4C) exhibited negative charge (−62.9 mV at pH 6.3) in the aqueous medium is related to the results by Wallace *et al*.^67^. The negative charge of GO again confirmed the presence of carboxylic acid and hydroxyl groups. Nevertheless, after functionalization (Figure 4D) the nanomaterial exhibited zeta potential value of −2.4 mV (pH 6.3). The reduction in zeta potential could be attributed to the existence of evenly distributed zwitterionicpoly(sulfobetaine) (PSBMA). This trend was comparable to the results reported by Ulbricht group^68^. Further, the presence of all elements in the nanohybrid was confirmed in EDX and elemental mapping confirmed the uniform distribution of all the elements (Figure 5).

### Morphology of HF membranes

The cross-section images of the pristine PSF HF membrane and nanocomposite HF membranes with various concentrations of GO@poly(SBMA-co-MBAAm) are presented in Figure S3, Supporting Information. It was found that the as-prepared HF membranes demonstrated typical asymmetric structure with sponge-like intermediate layer sandwiched between the top and bottom finger-like macro-voids. Since the coagulant (water) was intruded from both the top and bottom of the membrane, finger-like macro-voids were extended up to the sponge-like dense layer. Furthermore, as shown in Figure S3, Supporting Information, with increased concentration of GO@poly(SBMA-co-MBAAm) the porosity was gradually increased (TM-1 to TM-2) compared to TM-0 membrane. However, TM-3 membrane with 0.5 wt% of GO@poly(SBMA-co-MBAAm) demonstrated reduced porosity. The reduced porosity was attributed to the agglomeration of GO@poly(SBMA-co-MBAAm) at higher concentrations, which blocked some of the membrane pores.

### XPS analysis

**Figure 6:**
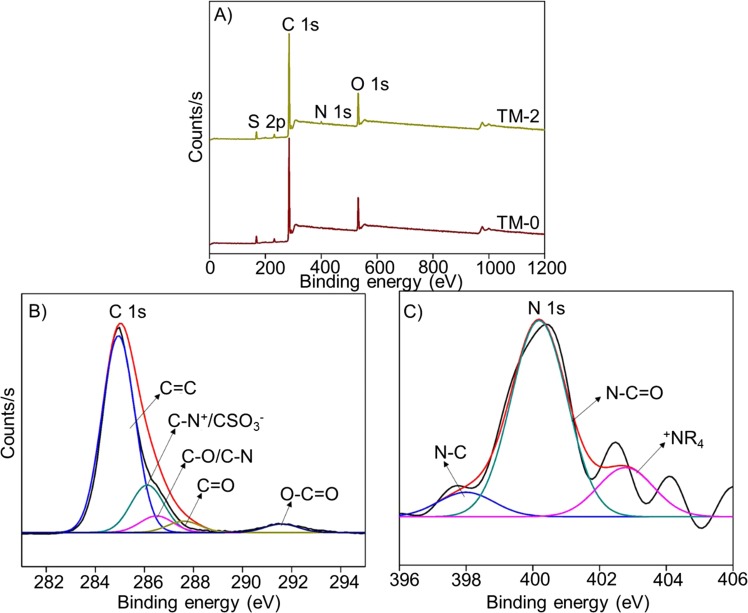
(**A**) XPS wide-scan TM-0 and TM-2 membrane spectra, (**B**) high resolution C 1s and (**C**) N 1s spectra of TM-2 membrane.

Figure 6A depicts the wide range XPS spectra of both TM-0 and TM-2 membrane. For the TM-2 membrane, the peak at 284.98 eV corresponds to C 1 s element. O 1 s element peak detected at 532.48 eV. The peak at 167.98 eV can be ascribed to S 2p element. The new peak at 400.48 eV was due to the existence of N 1 s element, which was not observed in the pristine membrane (TM-0). Further, the C 1 s and N 1 s elemental peaks were deconvoluted and depicted in Figure 6B and C. In Figure 6B,C 1 s was deconvoluted into five different peaks with binding energies of 284.9, 286.1, 286.3, 287.6 and 291.58 eV, which are assigned to C-C/C=C, CN^+^/CSO_3_^−^_,_ C-O/C-N, C=O, and O-C=O respectively. In Fig. 6C, N 1 s was deconvoluted into three peaks namely N-C=O and N-C and R_4_N^+^with the binding energies of 400.48, 398.18 and 402.28 eV. The atomic % of all the elements present in the membrane are as follows. For TM-0 membrane, C - 83.10%, O - 12.21% and S - 4.69% and for TM-2 membrane, C - 81.85%, O - 13.88%, S - 2.84% and N - 1.44%. Therefore, XPS analysis confirms the occurrence of nanohybrid in the membrane matrix.

### Surface properties

As tabulated in Table S3, with an increase in the concentration of nanohybrid, porosity was also increased related to the pristine membrane. As shown, the TM-2 membrane exhibited an increased porosity of 61.1 ± 0.34%. Conversely, the TM-3 membrane shown the reduced porosity of 57.8  ± 0.52%. The reduced porosity was attributed to the agglomeration of nanohybrid at a higher concentration. The obtained result is well aligned with the FESEM cross-sectional morphology of the membranes (Figure S3, Supporting Information).

The hydrophilicity of the membrane was measured by contact angle analysis. In general, the hydrophilic membrane surface is less prone to fouling^69,70^, which is necessary for reducing the maintenance cost and to increase the membrane lifecycle. Figure S4 in Supporting Information depicts the contact angle of membranes. The contact angle of the pristine membrane (TM-0) was 80 ± 1.2°, however, in the case of the TM-2membrane, the contact angle was 68 ± 1.5°. The reduction in contact angle could be ascribed to the addition of hydrophilic GO@poly(SBMA-co-MBAAm). The presence of both anionic and cationic functional groups in SBMA, render more affinity to bind with “free water” molecules, which results in reduced contact angle. Further, during the phase inversion, the added nanomaterials tend to transfer towards the top layer to reduce the interfacial energy. In contrast, while increasing the nanomaterial concentration from 0.25 to 0.5 wt%, the contact angle was increased to 70 ± 1° due to the presence of hydrophobic alkyl functional groups in functionalized GO and reduced porosity of the membrane^71^.

To understand the surface hydrophilicity further, water uptake capacity of the pristine and nanocomposite membrane was carried out and presented in Table S3. The nanocomposite membrane TM-2 exhibited the highest water uptake capacity of 60.3 ± 1.8% compared to the TM-0 membrane of 39.6 ± 1.4%. Conversely, for the TM-3 membrane, it was reduced to 57.1 ± 1.5% as hydrophilicity was reduced.The obtained result is in good agreement with the contact angle and porosity results.

The PXRD diffraction patterns of GO, GO@poly(SBMA-co-MBAAm) and TM-2 membrane are presented in Figure S5, Supporting Information. The PXRD pattern of TM-2 membrane exhibited a wide, weak diffraction peak at 2θ value of ~17° indicating an amorphous structure of PSF^72^. As explained above (Figure 4A), the [001] reflection of GO at 2θ = 9.8° was not observed in the TM-2 membrane as the regular stack of GO was demolished due to the intercalation of nanosize poly(SBMA-co-MBAAm).

The surface zeta potential of TM-0 and TM-2 T-UF HF membranes was analyzed as a function of pH and presented in Figure S6, Supporting Information. It was shown that, both the membranes exhibited a negative charge above pH 3.3. The isoelectric point (IEP) of TM-0 and TM-2 membranes was observed at pH 3.06 and 2.62. Furthermore, the TM-2 membrane demonstrated an increased negative charge compared to the TM-0 membrane. The slightly increased negative charge, attributed to the added negatively charged GO@poly(SBMA-co-MBAAm) nanomaterial.

The MWCO of all the T-UF HF membranes was determined from the rejection of PEG with different molecular weights. As can be seen from Figure S7, Supporting Information while increasing the molecular weight of the PEG, the rejection percentage also increases correspondingly. TM-0 HF membrane exhibited slightly higher MWCO than other membranes. However, all the T-UF HF nanocomposite membranes exhibited almost the same MWCO. Especially, the MWCO of the TM-2 membrane was 10665 Da and Stokes radius of PEG solute was 2.93 nm respectively, which confirmed that the as-prepared membrane is ultrafiltration membrane.

### Thermal stability

The effect of GO@poly(SBMA-co-MBAAm) nanomaterial on the thermal stability of the PSF HF membrane was analyzed by TGA. As shown in Figure S8, Supporting Information, the TM-2 membrane exhibited weight loss below 230 °C due to water and CO_2_ release. The weight loss of around 500 °C in TM-0 and TM-2 HF membranes was attributed to the decomposition of the aromatic backbone in PSF.

### Pure water flux study

The pure water flux (PWF) of as-prepared membranes is represented in Table S3. In general, PWF is mainly associated with the hydrophilicity and slack structure of nanocomposite membranes. It was observed that PWF was enhanced due to the incorporation of GO@poly(SBMA-co-MBAAm) nanohybrid, which was coherent with the obtained results of contact angle and water uptake. To a more precise, pristine membrane (TM-0) revealed the PWF of 22.5 L/m^2^h. However, in the case of the TM-2 membrane, PWF was improved to 49.6 L/m^2^h. This enhancement was attributed to the incorporation of nanohybrid, which provided improved hydrophilicity, porosity and slack structure of the nanocomposite membrane. The zwitterionic materials have a functional group such as sulfonate and a quaternary ammonium group, which has a strong attraction towards the water molecules via electrostatic attraction. Therefore, the attraction between the water molecules and the membrane surface will be more. Further, the incorporated nanohybrid in the polymer matrix can diminish the interaction of polymer chains to some extent. Therefore, it decreases the resistance to the transport of water molecules across the membrane and led to increased flux. Nevertheless, for the TM-3 membrane the PWF, hydrophilicity and porosity were decreased. The reason for this reduction is as follows. With a further increase of nanohybrid, tend to agglomerate and obstruct the pathway of permeation. As a result, PWF and porosity were reduced. The decreased hydrophilicity was due to the enhancement of hydrophobic alkyl groups in the MBAAm and SBMA. In summary, the as-prepared T-UF HF membrane noticeably exhibited an increased PWF compared to the pristine membrane.

### Dye separation performance of the membrane

#### Effect of dye concentration

**Figure 7:**
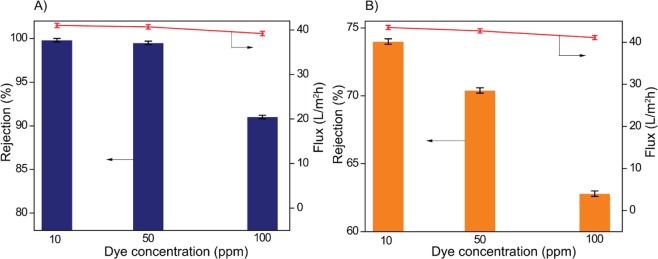
TM-2 membrane performance as a function of dye concentration for (**A**) RB-5 and (**B**) RO-16 (1 bar and pH 7).

**Figure 8:**
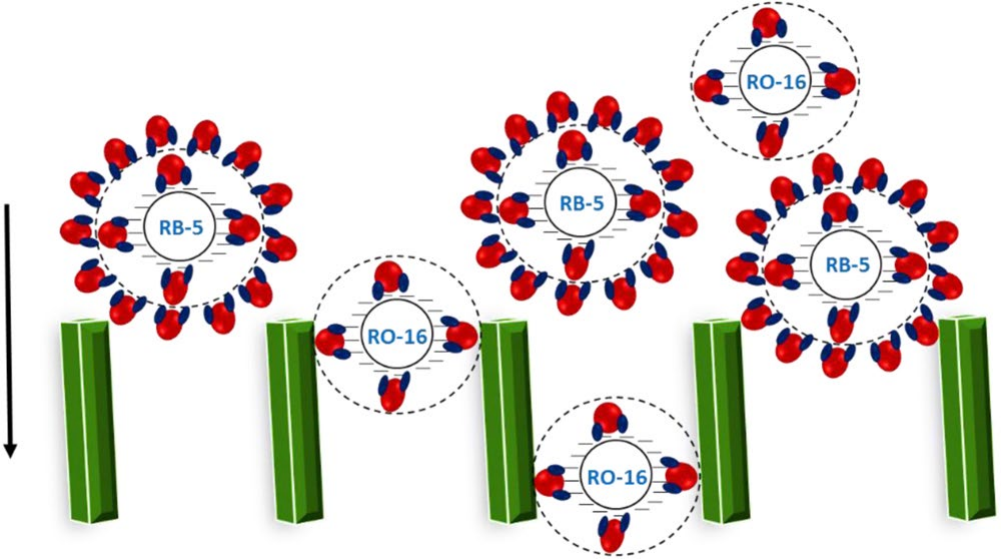
Schematic representation of hydration of dye molecules.

To evaluate the dye removal capacity of the as-prepared membrane, the rejection experiment was carried out with various concentrations of RB-5 and RO-16. Among all the prepared membranes, TM-2 was preferred for the dye removal studies. Figure 7 presents the percentage of dye rejection with dye solution flux as a function of dye concentration. As it is clear from Figure 7, the membrane demonstrated the highest rejection of 99% for RB-5 and 74% of RO-16 at 10 ppm of dye concentration. However, for both dyes, the dye rejection and dye solution flux were decreased with the increase of dye concentration, which is in accordance with the results reported by Liu *et al*.^30^. The four charged RB-5 demonstrated the highest rejection than the two charged RO-16. In general, the higher the charge of the dye molecules, the greater would be the degree of hydration. Consequently, the size of the dye molecule increases. As shown in Figure 8, the greater hydration degree and higher molecular weight of RB-5 could be attributed to the higher rejection compared to low molecular weight and less hydrated RO-16. Furthermore, the reason for decreased rejection was due to the increased concentration of impurities such as Cl^−^, SO_4_^2−^ and HCO_3_^−^ in the feed solution. The presence of such ionic species would increase the electrostatic shielding effect, thereby decreasing the rejection of dyes^12^. A similar report had been published for the decreased rejection of direct red 23^28^. Meanwhile, the permeated dye molecules cause fouling, which results in decreased dye solution flux slightly.

#### Effect of pressure

**Figure 9:**
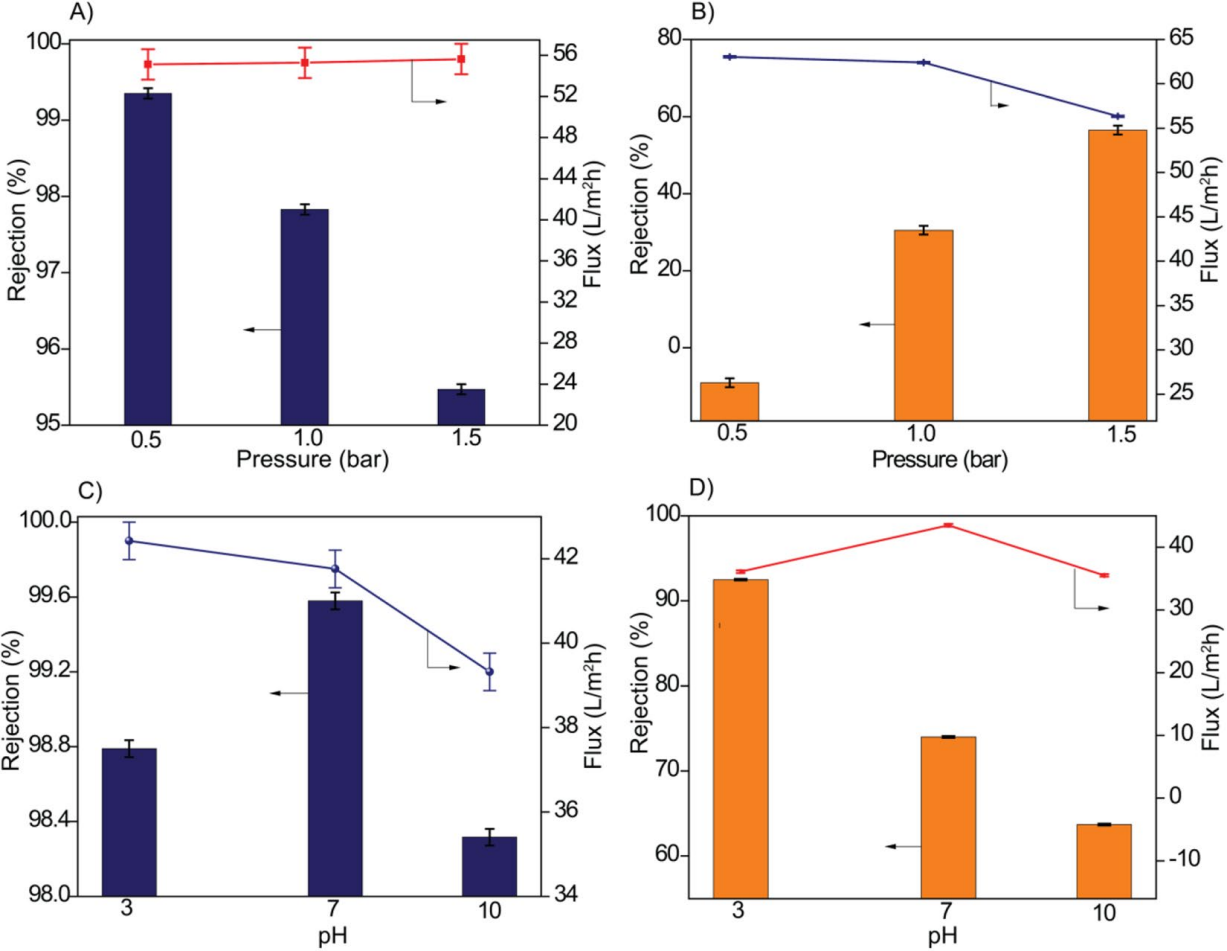
TM-2 membrane performance as a function of pressure for (**A**) RB-5 and (**B**) RO-16 (10 ppm and pH 7) and as a function of pH (**C**) RB-5 and (**D**) RO-16 (10 ppm and 1 bar).

The performance of the TM-2 membrane with respect to applied pressure is depicted in Figure 9A and B. Dye solution flux increases with the increase of pressure for both the dyes, however, dye rejection was decreased. The decreased rejection could be attributed to the enhancement of concentration polarization effect, which improves the permeation of dye molecules. The observed results are in parallel with the results reported by Lin *et al*.^28^ and Petrinic *et al*.^73^.

#### Effect of feed solution pH

The separation efficiency of membranes can be effectually altered by the solution pH since the electrochemical properties of the membrane surface and the dye molecules are directly affected. Figure 9C and D describe the effect of solution pH on dye rejection and dye solution flux. The optimum state of greater rejection and flux was observed at pH 7. However, at pH 10 both rejection and flux were declined. In the case of basic condition, the membrane surface is highly ionized and undergoes severe swelling. The swelling could effectively make the membrane surface (skin layer) thicker^74^, therefore, membrane flux decreases. Furthermore, the decreased rejection can be ascribed to the inferior electrostatic repulsion between the membrane surface and dyes. At high basic conditions, the electrostatic interaction is screened by the presence of OH^–^ions. It was noted that at pH 3, dye rejection was increased, and flux was declined. In acidic conditions, dye molecules get protonated to a greater extent, which in turn solubility of dye molecules are reduced and get precipitated in the feed tank. As a result, rejection increases owing to precipitation, while flux decreases because of membrane fouling from dye precipitation on the membrane surface. The feed and permeate digital photographic images of RB-5 and RO-16 is depicted in Figure S9, Supporting Information.

#### Salt and salt/dye mixture filtration

As textile wastewater is a mixture of dye and inorganic salts, it is important to study the rejection profile of salt and salt/dye mixture. Further, the added salt has a direct impact on membrane performances^75–77^. Subsequently, a membrane with high rejection of dye molecules with high salt permeation is highly needed to process the textile wastewater. TM-2 membrane exhibited complete permeability to NaCl and <5% rejection to Na_2_SO_4_. The filtration performance of the TM-2 membrane for RB-5 and RO-16 at different concentrations of Na_2_SO_4_ is illustrated in Figure S10Aand B, Supporting Information. As reported by Nilsson *et al*. salt/dye solution flux was decreased with the increase of salt concentration. The reduced flux was attributed to the change in the osmotic pressure, which reduces the net driving pressure. In spite of the TM-2 membrane exhibits lower rejection for Na_2_SO_4_, water transports faster than salt (“Dilute effect”) due to steric exclusion^78^. However, the rejection was increased as the dye molecules form aggregates under the influence of added salt. Some of the recent literature reported and commercially available membranes are summarized in Table S4.

Furthermore, a short-term stability study was performed and depicted in Figure S11, Supporting Information. During the 24 h of filtration, there was a slight decrease in the dye solution flux and a small increment in the rejection was observed. The change in the rejection and flux was ascribed to the formation of the dye cake layer and cake-enhanced concentration polarization.

#### Antifouling study

The membrane filtration performance greatly depends on the antifouling property. Typically, membranes used in wastewater treatment are more vulnerable to membrane fouling, which was ascribed to the adsorption and aggregation of foulants on the hydrophobic membrane surface. Consequently, membrane flux is reduced. As the foulants are contacting the membrane surface via hydrogen bonding, electrostatic interaction and weak van der Walls force^79^, simple hydraulic cleanings will not help to recover the maximum membrane flux until the membrane has antifouling property. Hence, high flux, low fouling tendency, and high rejection capability are the prerequisites of the nanocomposite membrane. In the present study, BSA was used as a model protein. Generally, as the size of BSA molecules is larger than the membrane pore size, it adsorbs on the membrane surface rather than penetrate the pore. Thus, it causes severe fouling on the membrane surface.

Figure S12, Supporting Information signifies the water flux before and after BSA filtration. The water flux was reduced to a greater extent after BSA filtration, which was attributed to the blockage of membrane pores by adsorption of BSA molecules on the membrane surface and concentration polarization. However, after washing, the nanocomposite membrane could recover the maximum water flux while compared to the pristine (TM-0) membrane.

The calculation of the flux recovery ratio (FRR) would be useful to determine the antifouling performance of the membranes. In addition, total organic fouling (R_t_), reversible fouling (R_r_) and irreversible fouling (R_ir_) were also calculated for better understanding and values are tabulated in Table S3. The TM-2 membrane showed the FRR of 73.9% compared to the TM-0 membrane of 41.2%. The enhanced FRR of the nanocomposite membrane was owing to the improved hydrophilicity and surface charge by the addition of GO@poly(SBMA-co-MBAAm) nanohybrid. The better hydrophilicity of the nanocomposite membrane assists to form a hydration layer on the membrane surface, which hinders the adsorption of protein molecules^80^. Thus, the loosely adsorbed foulants were easily removed with a simple hydraulic water wash. Moreover, since the as-prepared nanocomposite membrane is negatively charged at pH 7.4, it also would help to inhibit the adsorption of negatively charged BSA molecules through electrostatic repulsion. The *R*_*t*_ of the pristine membrane was 81.8%, which shows the inferior antifouling nature of TM-0. The nanocomposite membrane TM-2 demonstrated the *R*_*ir*_ of 17.4%, which confirmed the loose adhesion of foulants on the membrane surface. In the case of TM-0, *R*_*ir*_ was 38.8% as the foulants had a strong attraction towards the membrane surface. Still, TM-3 membrane performance was less compared to TM-2. The reason for the reduced performance of TM-3 could be attributed to the agglomeration of nanohybrid at the higher concentration. In summary, the antifouling property of the TM-2 membrane was significantly enriched.

## Conclusions

The prepared GO surface was functionalized with zwitterionic sulfobetaine via DPP. The as-synthesized nanohybrid was incorporated into the PSF membrane matrix and tight ultrafiltration hollow fiber membranes were prepared by dry/wet phase inversion method. The modification of GO was confirmed by spectroscopic and microscopic techniques. The existence of nanohybrid in the membrane matrix was investigated by XPS analysis. The impact of nanohybrid on the hydrophilicity, water uptake, porosity, MWCO, morphology, flux, and antifouling capacity of nanocomposite membranes were explored. The nanocomposite membrane exhibited high pure water flux (49.6 L/m^2^h) and reactive dyes such as RB-5 (99%) and RO-16 (74%) rejection with high salt flux, which was owing to the presence of nanohybrid. TM-2 membrane with 0.25 wt% loading of nanohybrid exhibited the *R*_*ir*_ of 17.4%, signifying enhanced antifouling ability; the as-prepared novel tight UF hollow fiber membrane demonstrates a potential application for salt/dye mixture separation in textile wastewater.

## Supplementary infromation

Supplementary information.
