# Supplementary material for: One-step synthesis of zwitterionic graphene oxide nanohybrid: Application to polysulfone tight ultrafiltration hollow fiber membrane
Source: Sci Rep. 2020 Apr 23;10:6880. doi: 10.1038/s41598-020-63356-2 (PMC7181782; doi:10.1038/s41598-020-63356-2)
Supplement: Supplementary file 1 — Supplementary information. [file 41598_2020_63356_MOESM1_ESM.docx]

**Supporting information**

**One-step synthesis of zwitterionic graphene oxide nanohybrid: Application to polysulfone tight ultrafiltration hollow fiber membrane**

G. P. Syed Ibrahim^a^, Arun M. Isloor^a*^, A. F. Ismail^b^ and Ramin Farnood^c^

^a^Membrane Technology Laboratory, Chemistry Department, National Institute of Technology

Karnataka, Surathkal, Mangalore 575 025, India.

^b^Advanced Membrane Technology Research Center (AMTEC), Universiti Teknologi Malaysia, 81310, Skudai, Johor Bahru, Malaysia.

^c^Department of Chemical Engineering and Applied Chemistry, University of Toronto,

Canada.

Corresponding author: [isloor@yahoo.com](mailto:isloor@yahoo.com), Ph : +919448523990

**Characterizations**

The morphology of GO@poly(SBMA-co-MBAAm) was examined by transmission electron microscopy (TEM - JEOL JEM-2100). The presence of all the elements in the nanohybrid was confirmed and elemental mapping was performed using energy dispersive X-ray (EDX, X-Max Oxford instruments) analysis. FT-IR spectra with a scanning range of 4000-600 cm^-1^ were measured using a BRUKER ALPHA ECO FT-IR spectrometer as KBr pellets. The thermal stability of the as-synthesized nanohybrid was determined by thermogravimetric analysis (TGA, HITACHI EXSTAR TG/DTA 6300). The nanocomposite was heated from room temperature to 800 ^o^C at a heating rate of 10 ^o^C min^-1^ under nitrogen gas. The polymorphism of GO and nanohybrid was recorded using powder X-ray diffractometer (PXRD, Rigaku Miniflex) in the range of 5 to 40^o^ using Cu Kα as an X-ray source. The surface charge of the nanohybrid was characterized using the HORIBA nanoparticle analyzer and membrane zeta potential was analyzed by Anton Paar electrokinetic analyzer (SurPASS). SEM (HITACHI TM3000) was used to observe the cross-section images of the membranes. Surface hydrophilicity was analyzed by the static water contact angle, which was performed in contact angle goniometer (Data Physics instruments, OCA20). The membrane elemental composition and presence of nanohybrid were studied by X-ray photoelectron spectroscopy (XPS, THERMO FISHER Scientific K-ALPHA) analysis. Al Kα radiation (1486.6 eV) was employed as an X-ray source and the take-off angle was 90^o^.

~ 0.5 g of membrane sample was taken and both the ends were sealed using epoxy resin followed by drying at 50 ^o^C until the constant weight. The sample was submerged in distilled water for 24 h. Subsequently, wet weight (*W_w_*) was noted after mopping with blotting paper to remove the adsorbed water on the membrane surface. Lastly, the membrane was dried completely and dry weight (*W_d_*) was noted. The % water uptake was calculated by the following equation (1).

$\% water uptake = \frac{W_{w}-W_{d}}{W_{w}} \times100$ (1)

The porosity (ε) of the membranes was determined gravimetrically as defined in the below equation (2).

ε (%) = $\frac{W_{w}- W_{d}}{A\times l\times p}$ × 100 (2)

Where ‘*W_w_*’ and ‘*W_d_*’ are the wet and dry weight of the membrane, ‘*A*’ is the area of the membrane (cm^2^),$'p'$ is the density of pure water (0.998 g cm^-3^) and $'l'$ is the thickness of the membrane (cm).

**Permeation studies**

The T-UF HF membranes were characterized by measuring the pure water flux (PWF), salt and dye rejection using a lab-scale cross-flow module at RT. In each module, five numbers of HF membranes with 10 cm of length were potted in stainless steel adaptor using epoxy resin, which allowed to dry at room temperature for 24 h. The permeation and rejection studies were carried out at 1 bar pressure. In order to reach a steady-state, all the membranes were compacted for 35 min at 2 bar before testing. Later PWF, *J_w_* (L/m^2^h) was calculated using the below equation (3).

$J_{w}= \frac{Q}{\Delta t\times A}$ (3)

Where ‘*Q*’ is the volume of permeated water (L), ‘*A*’ is the effective area of hollow fiber membrane (m^2^) and ‘*∆t*’ is the permeation time (h). The rejection experiments were conducted using a different concentration of RB-5 and RO-16. The concentration of salts was measured from conductivity (JENWAY 4520 conductivity meter) analysis and concentration of dye molecules was measured using UV-Vis spectrophotometer (DR6000 HACH). The percentage rejection, ‘*R*’ was determined using this equation (4).

$R (\%)= \frac{C_{f}-C_{p}}{C_{f}} \times100$ (4)

Where ‘*C_p_*’ and ‘*C_f_*’ are the solute concentration in permeate and feed respectively.

The molecular weight cut-off (MWCO) of the membrane was characterized by filtering sequence of polyethylene glycol (PEG) with a molecular weight of 2000, 4000, 6000 and 10000 Da at the concentration of 100 ppm at 1 bar. The PEG concentration in both feed and permeate was evaluated using a total organic carbon analyzer (TOC, SHIMADZU). The PEG rejection, ‘*R*’ was determined using equation 4. MWCO is the molecular weight of solute upon which at least 90% of rejection can be attained.[^1^](#_ENREF_1) Furthermore, the Stokes radius of PEG solute was determined [equation (5)] from its average molecular weight.[^2^](#_ENREF_2) Where ‘*r_p_*’ is in m and molecular weight (MW) in Da.

$r_{p}=16.73 X {10}^{-12}\times{MW}^{0.557}$ (5)

**Antifouling study**

The antifouling performance of the T-UF HF membrane was evaluated using 800 ppm BSA solution at pH 7.4 as feed at room temperature and 1 bar pressure. In brief, after PWF (*J_w1_*) study for 60 min, the feed tank was refilled with BSA solution and allowed for another 60 min filtration to calculate the BSA flux (J_p_). Subsequently, the membrane was washed with distilled water for another 60 min. Again PWF (*J_w2_*) of the cleaned membrane was measured. To assess the antifouling performance, flux recovery ratio (FRR) was measured using the below-mentioned equation (6).

$FRR (\%)= \frac{J_{w2}}{J_{w1}} \times100$ (6)

To understand the fouling process in detail, total organic fouling (*R_t_*, the degree of total flux drop owing to total fouling), reversible fouling (*R_r_*, fouling due to concentration polarization) and irreversible fouling (*R_ir_*, fouling due to adsorption of protein molecules) were also studied by below equations (7, 8 and 9).

$R_{t}\left( \% \right)= \left( 1-\frac{J_{p}}{J_{w1}} \right) \times100$ (7)

$R_{r}\left( \% \right)= \left( \frac{J_{w2}-J_{p}}{J_{w1}} \right) \times100$ (8)

$R_{ir}\left( \% \right)= \left( \frac{J_{w1}-J_{w2}}{J_{w1}} \right)\times100$ (9)


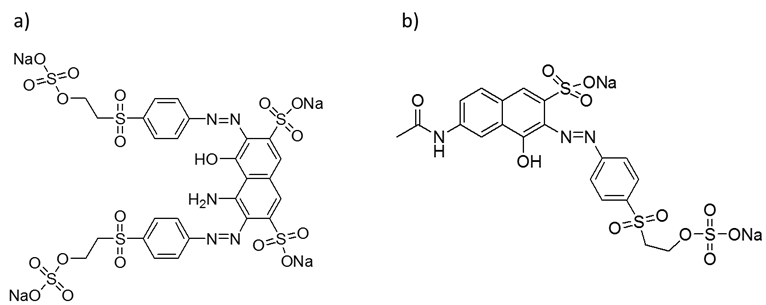


**Figure S1.** Chemical structure of (a) RB-5 (MW = 991.82 Da) and (b) RO-16 (MW = 617.54 Da).


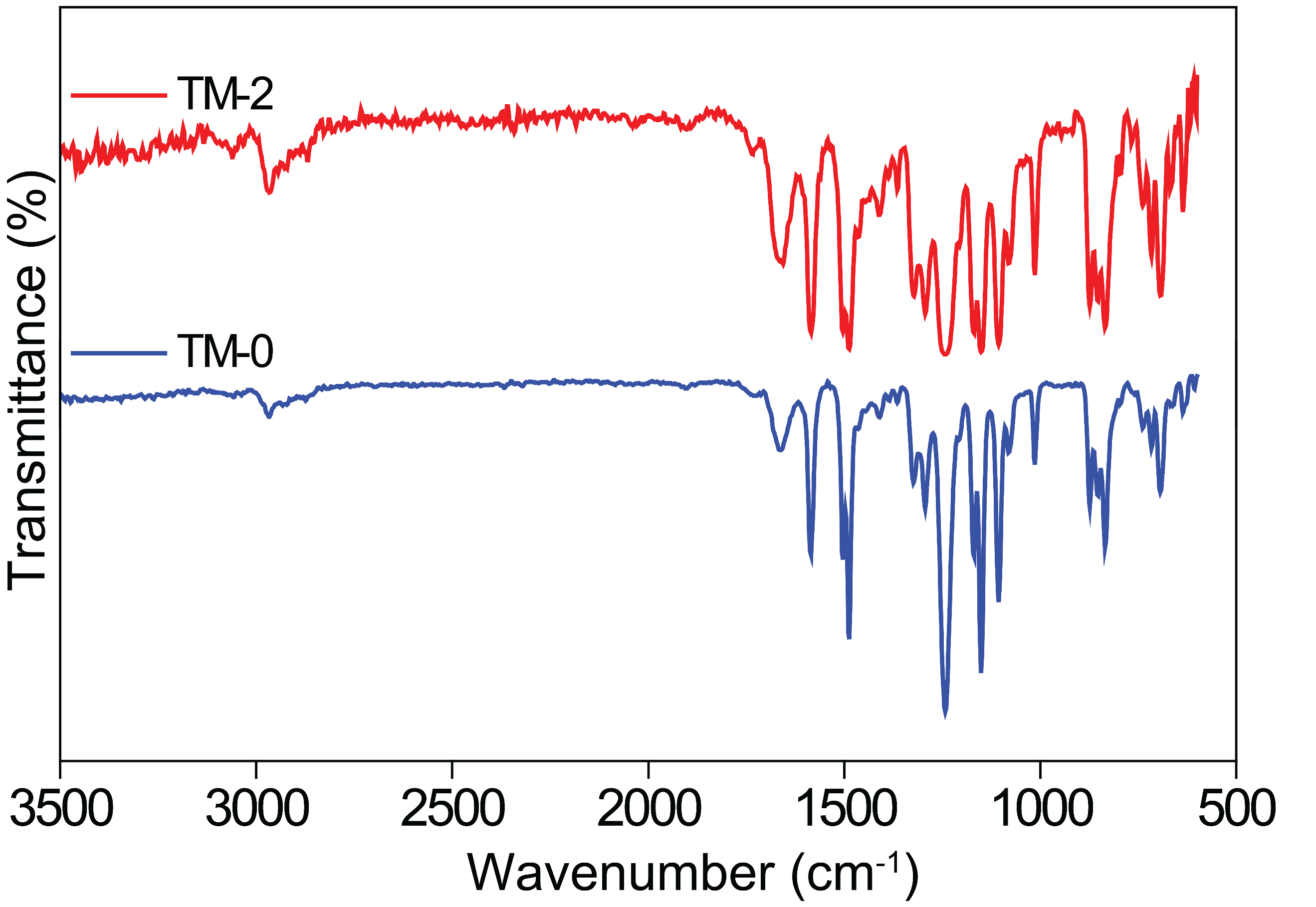


**Figure S2**. ATR-FTIR spectra of TM-0 and TM-2 membranes.


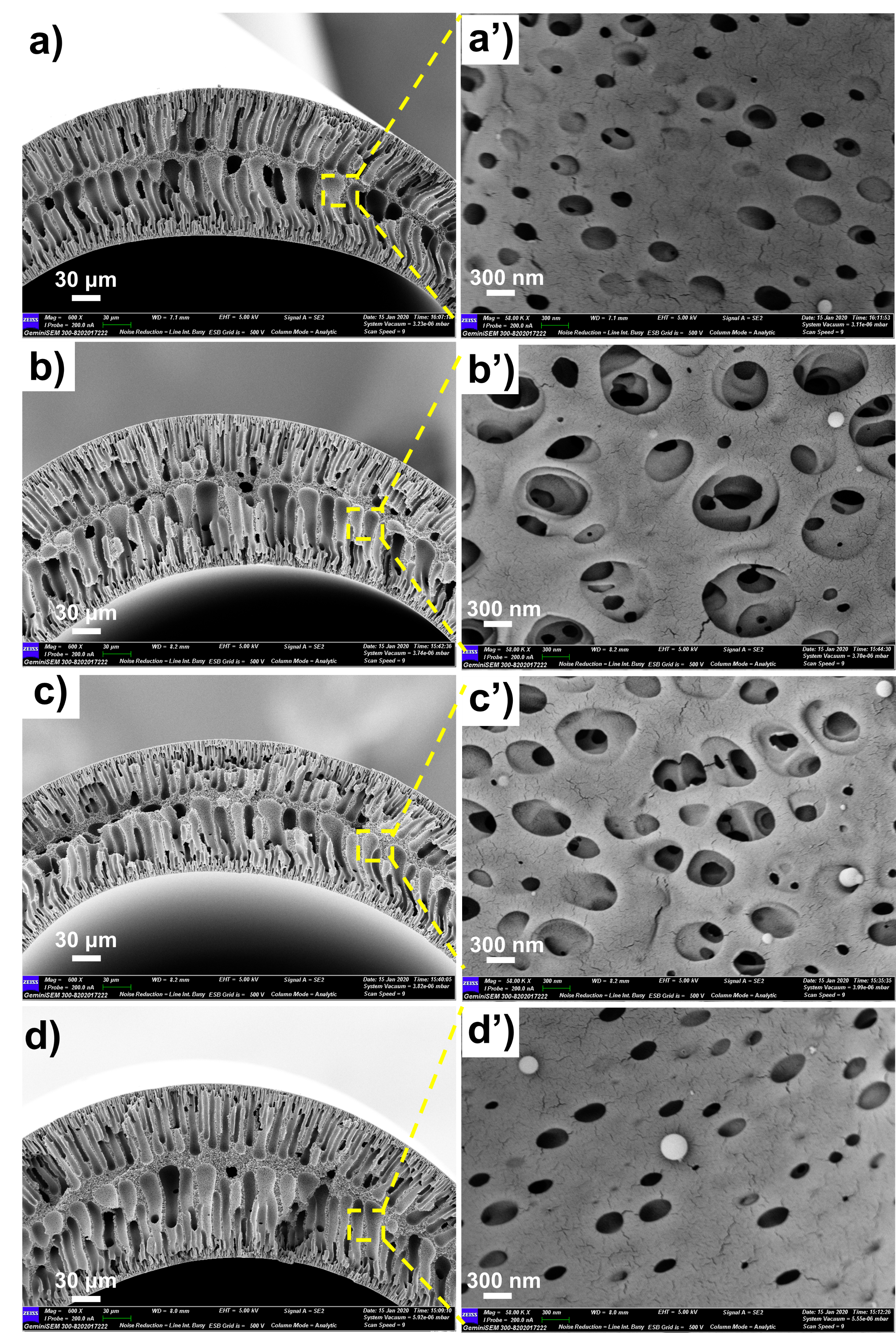


**Figure S3.** FESEM cross-sectional images of a), a’) TM-0, b), b’) TM-1, c), c’) TM-2 and d), d’) TM-3 membranes with different magnification.


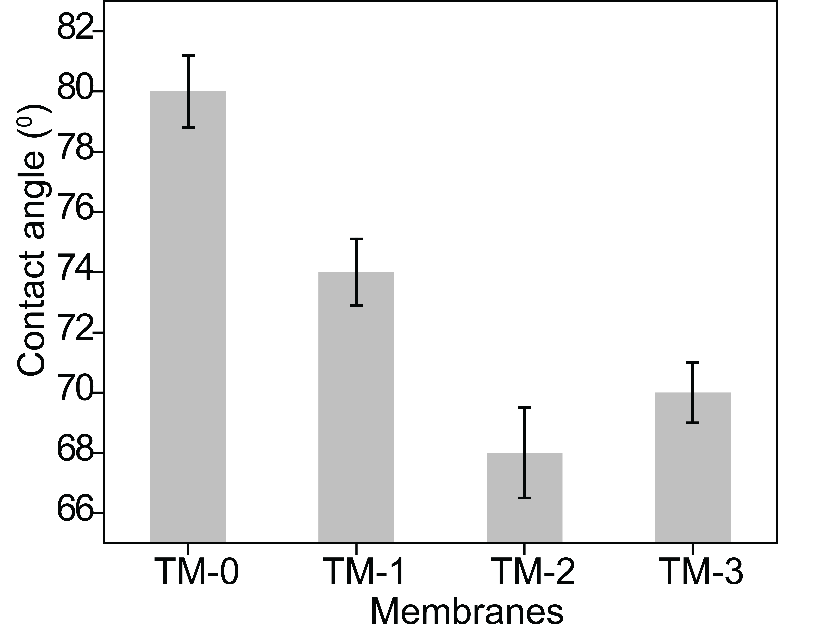


**Figure S4.** Contact angle of hollow fiber membranes.


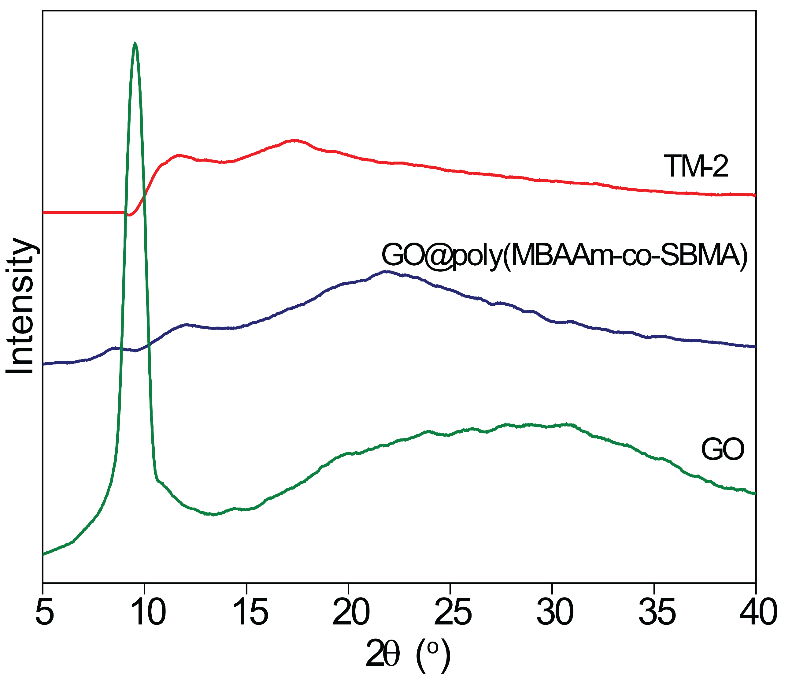


**Figure S5**. PXRD of GO, GO@poly(SBMA-co-MBAAm) and TM-2 membrane.


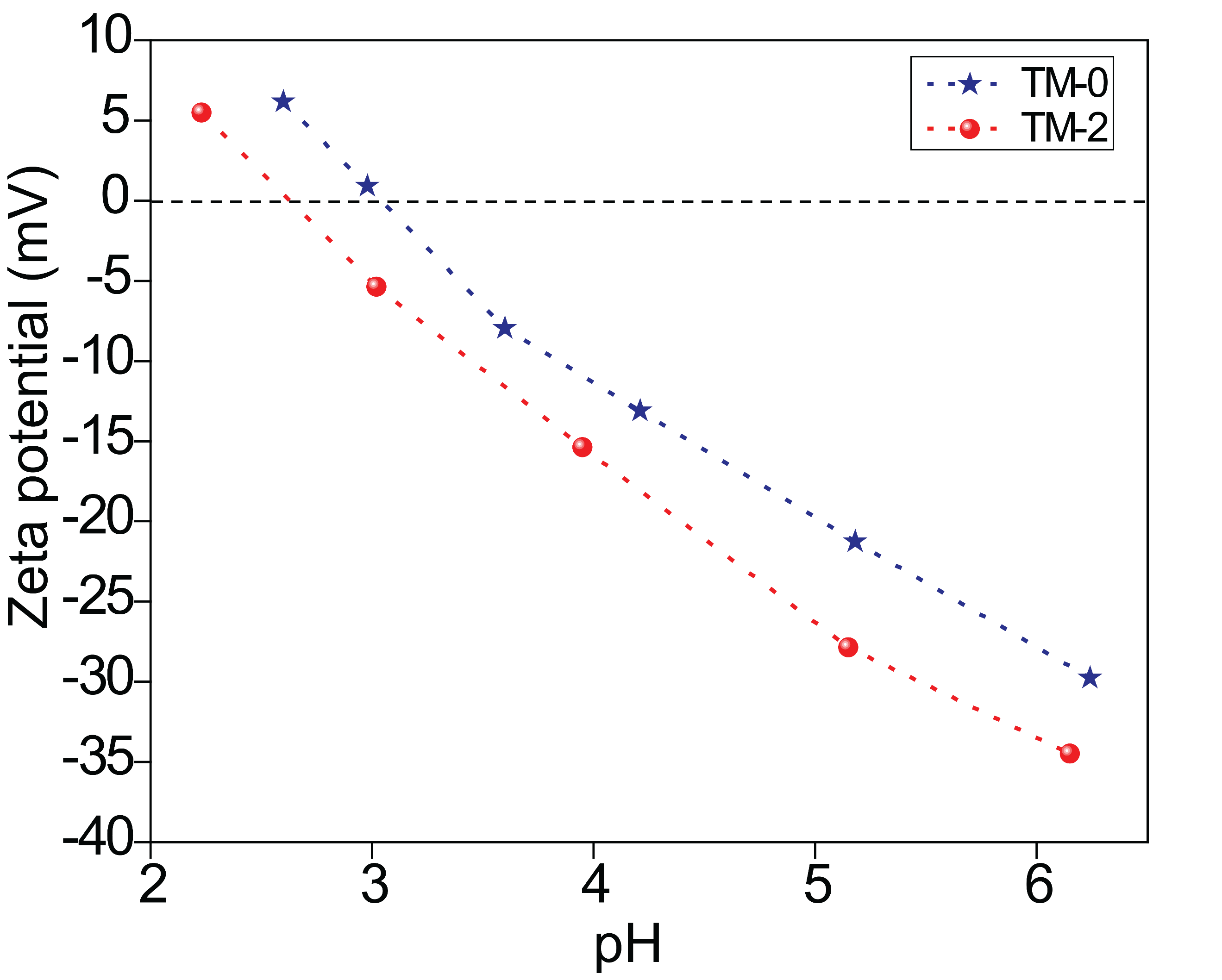


**Figure S6**. Zeta potential of hollow fiber membranes as a function of pH.


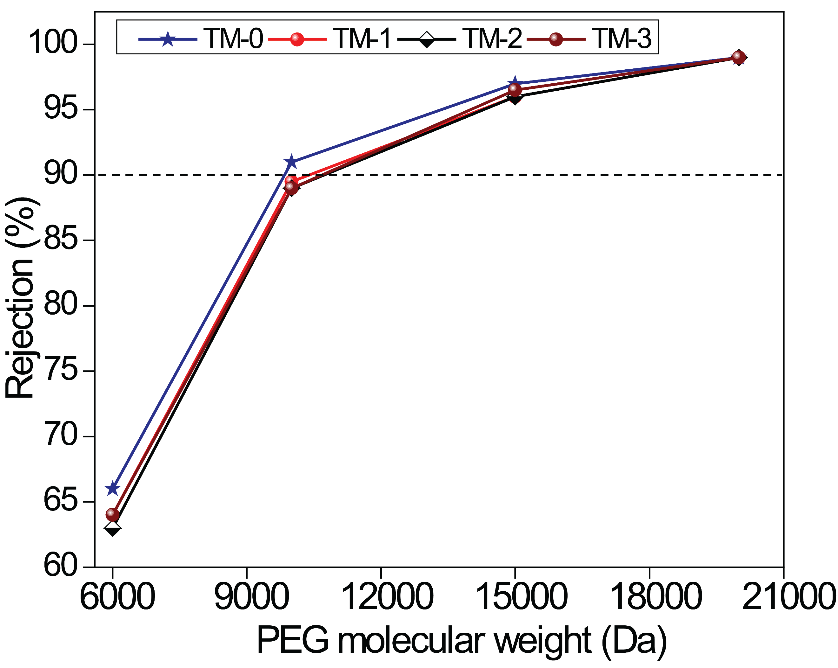


**Figure S7**. MWCO curve of hollow fiber membranes.


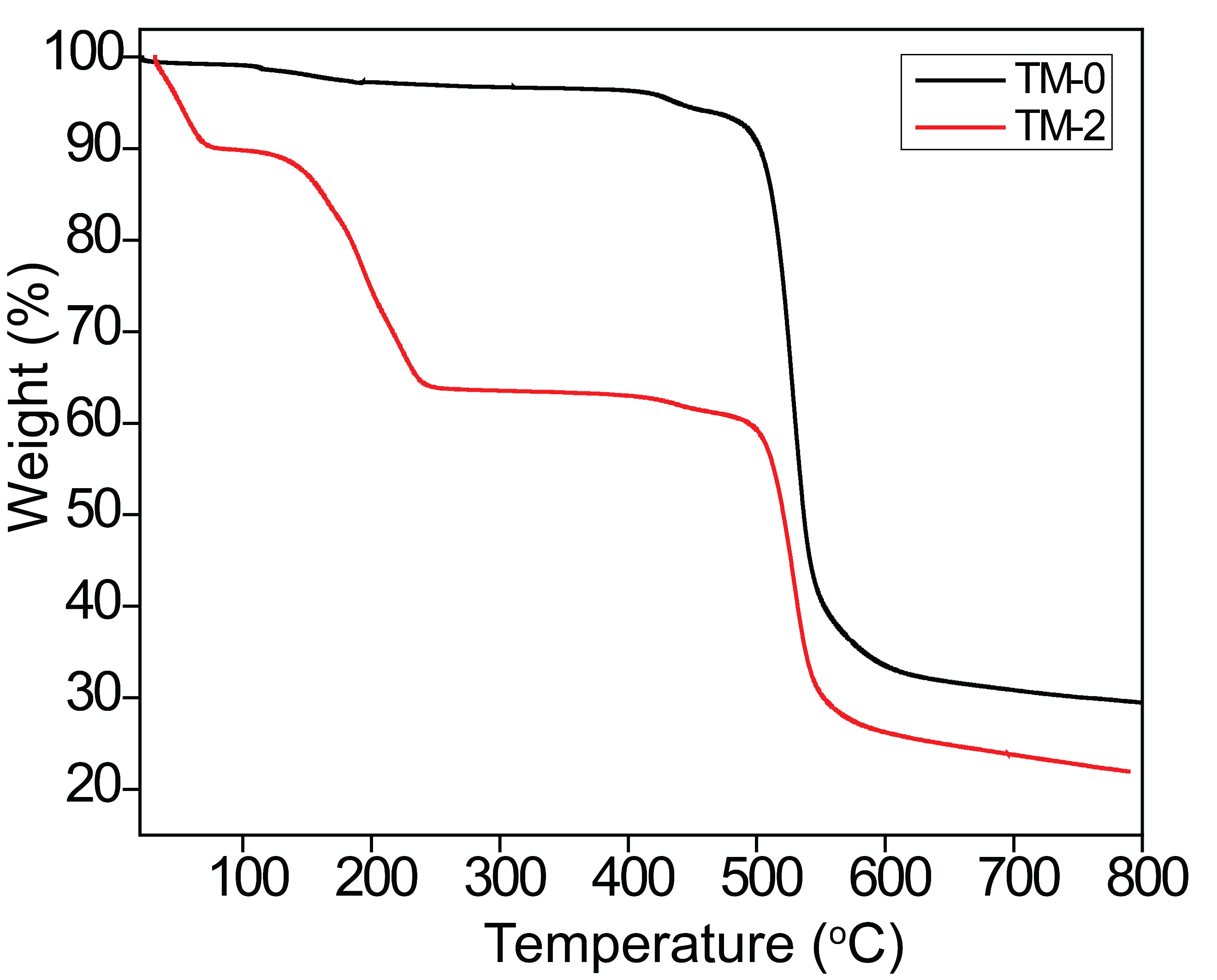


**Figure S8**. TGA curves of TM-0 and TM-2 membranes.


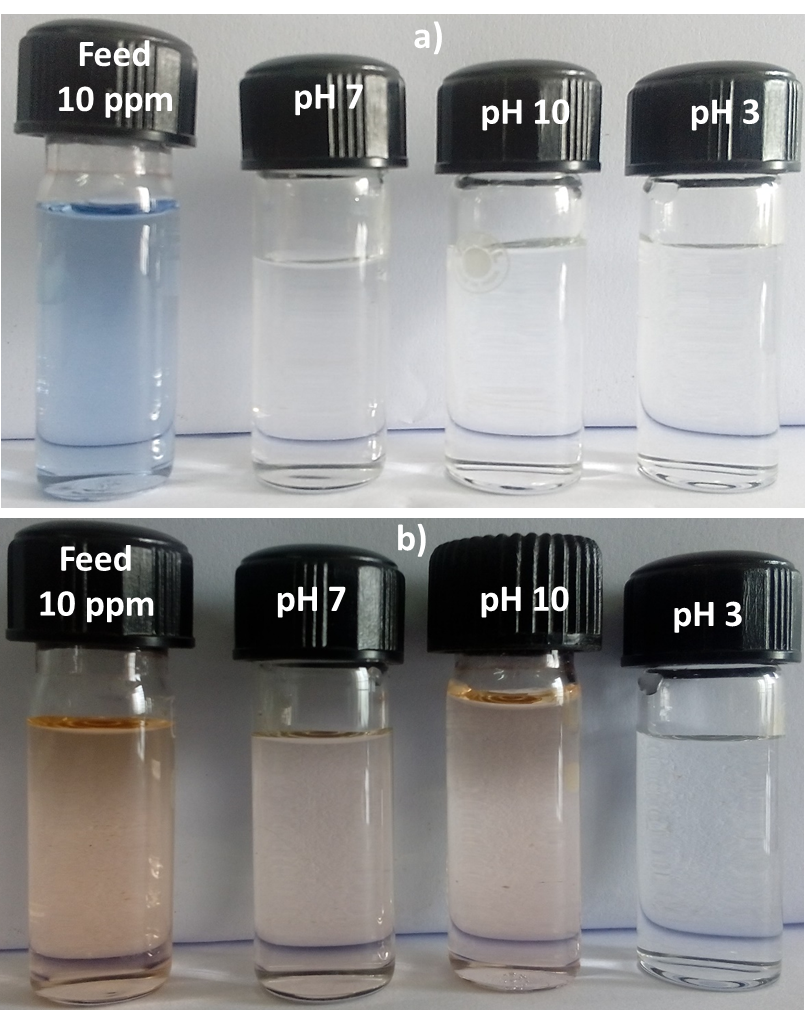


**Figure S9.** Digital photographic images of (a) RB-5 and (b) RO-16.


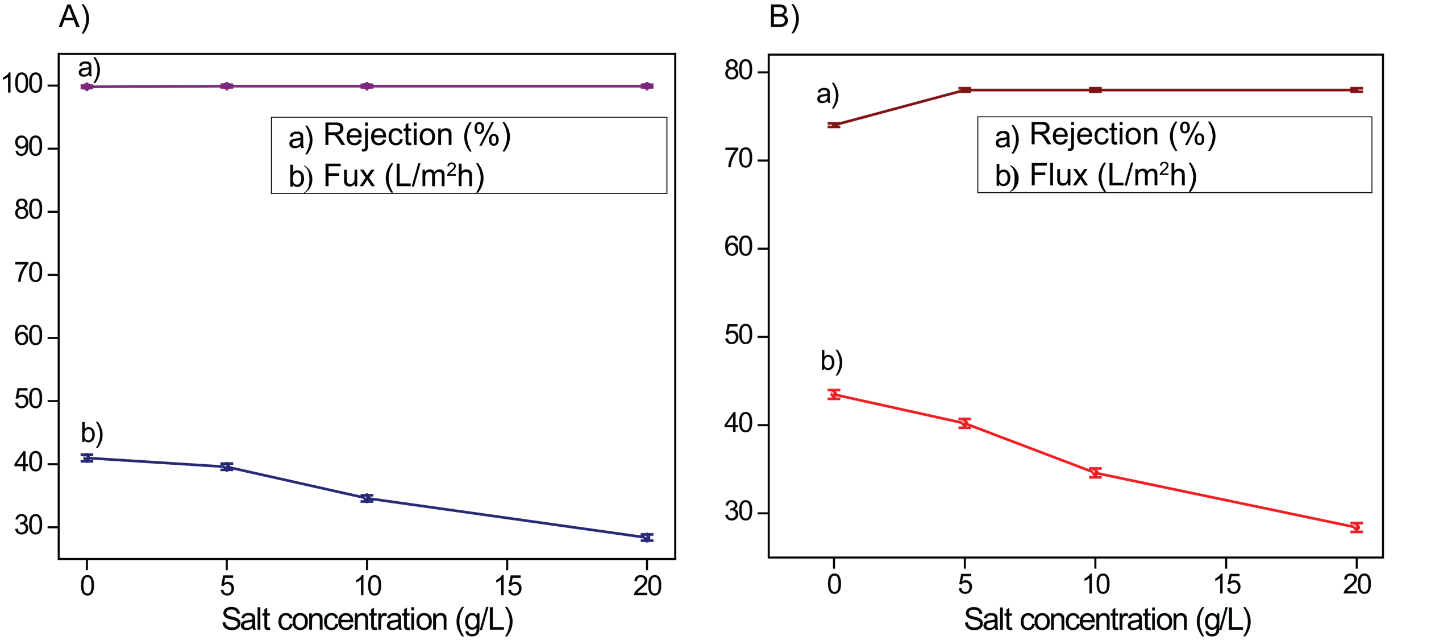


**Figure S10.** TM-2 membrane performance of salt/dye mixture for A) RB-5 and B) RO-16 (10 ppm of dye, 1 bar, and salt is Na_2_SO_4_).


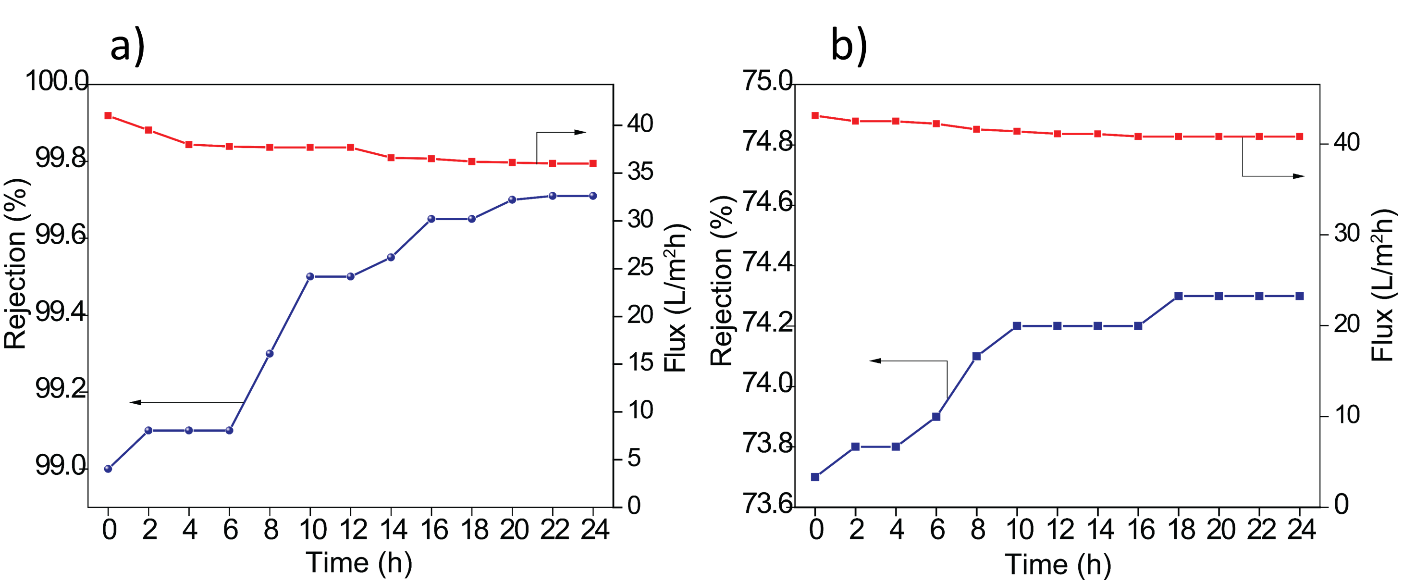


**Figure S11.** Short-term stability study of TM-2 membrane at 1 bar and 10 ppm, (a) RB-5 and (b) RO-16.


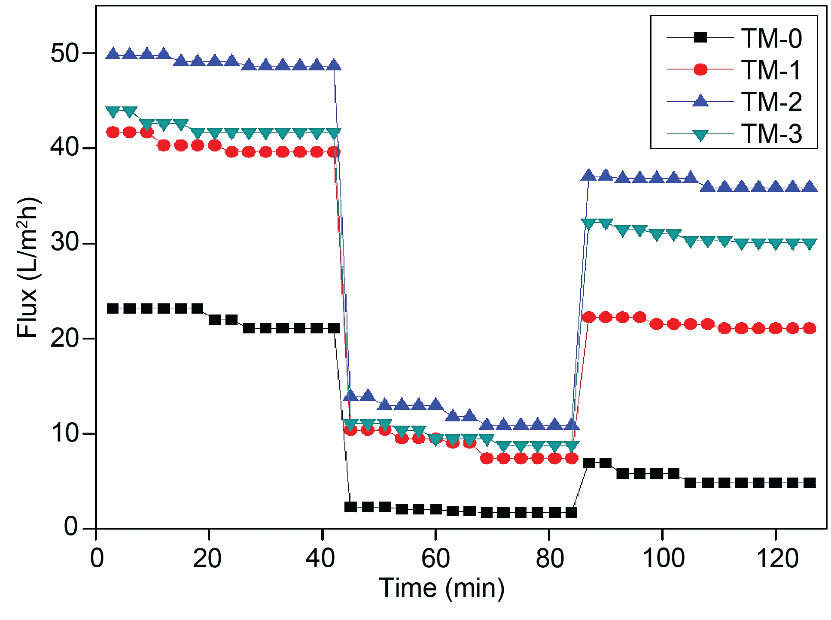


**Figure S12.** Water and BSA solution flux of pristine and nanocomposite membranes.

**Table S1.** Spinning parameters.

| Parameters | Conditions |
| --- | --- |
| Coagulation bath | Tap water |
| Spinneret (mm) | 1.1/0.55 (OD/ID) |
| Coagulation bath temperature (^o^C) | 27 |
| Bore flow rate (mL/min) | 2.5 |
| Dope extrusion rate (mL/min) | 3 |
| Bore fluid | Distilled water |
| Air gap (cm) | 1 |
| Collecting drum speed (RPM) | 7 |
| Humidity (%) | 60 |

**Table S2.** The composition of the dope solution.

| Membrane | PSF (g) | PVP (g) | NMP (g) | GO@poly(SBMA-co-MBAAm) (g) | GO@poly(SBMA-co-MBAAm) (wt%)* |
| --- | --- | --- | --- | --- | --- |
| TM-0 | 20 | 1 | 79 | 0 | 0 |
| TM-1 | 20 | 1 | 79 | 0.02 | 0.1 |
| TM-2 | 20 | 1 | 79 | 0.05 | 0.25 |
| TM-3 | 20 | 1 | 79 | 0.1 | 0.5 |

* With respect to PSF

**Table S3.** Properties of T-UF HF membranes

| Membrane | PWF  (L/m^2^ h) | Water uptake (%) | Porosity  (%) | Fouling (%) | | | |
| --- | --- | --- | --- | --- | --- | --- | --- |
|  |  |  |  | FRR | R_t_ | R_r_ | R_ir_ |
| TM-0 | 22.5 +0.67 | 39.6 +1.4 | 42.4 +0.42 | 41.2 +0.5 | 81.8 +0.23 | 21.2 +0.11 | 38.8 +0.34 |
| TM-1 | 40.3 +0.41 | 51.5 +2.1 | 49.7 +0.71 | 53.4 +0.45 | 76.3 +0.31 | 34.7 +0.32 | 24.1 +0.41 |
| TM-2 | 49.6 +0.54 | 60.3 +1.8 | 61.1 +0.34 | 73.9 +0.36 | 71.1 +0.33 | 39.1 +0.3 | 17.4 +0.38 |
| TM-3 | 42.1 +0.73 | 57.1 +1.5 | 57.8 +0.52 | 70.5 +0.71 | 72.7 +0.28 | 36.8 +0.43 | 20.7 +0.51 |

**Table S4.** Summary of reported literature and commercially available membranes for dye rejection.

| Membrane | Dye | *PWP (L/m^2^h) | **DSP (L/m^2^h) | Rejection  (%) | Ref. |
| --- | --- | --- | --- | --- | --- |
| GO-PSBMA/PES loose NF | Reactive red 49/reactive black 5 | 11.9 | 8.8 | 97.2/99.2 | [^3^](#_ENREF_3) |
| PAEK-COOH T-UF | Congo red | 29.5 | 25.0 | 99.8 | [^4^](#_ENREF_4) |
| Commercial Sepro NF 2A | Direct red 80 | 10.5 | 9.6 | 99.9 | [^5^](#_ENREF_5) |
| Commercial Sepro NF 6 | Direct red 80 | 13.7 | 13.2 | 99.9 | [^5^](#_ENREF_5) |
| UH004 (hydrophilic PES) | Direct red 80, direct red 23, and congo red | 27.5 | 27.0 | 98.9 | [^6^](#_ENREF_6) |
| GO@poly(SBMA-co-MBAAm)/PSF T-UF | Reactive black 5 / reactive orange 16 | 49 | 41/43.5 | 99.75/74 | Present study |

*PWF- Pure water permeability, ** DSF- Dye solution permeability.

**References**

[1] J. Mulder, Basic principles of membrane technology, Springer Science & Business Media, **2012**.

[2] J. Lin, C. Y. Tang, C. Huang, Y. P. Tang, W. Ye, J. Li, J. Shen, R. Van den Broeck, J. Van Impe, A. Volodin *J. Membr. Sci.* **2016**, *501*, 1-14.

[3] J. Zhu, M. Tian, J. Hou, J. Wang, J. Lin, Y. Zhang, J. Liu, B. Van der Bruggen *J. Mater. Chem. A*. **2016**, *4*, 1980-1990.

[4] C. Liu, H. Mao, J. Zheng, S. Zhang *J. Membr. Sci.* **2017**, *530*, 1-10.

[5] J. Lin, W. Ye, H. Zeng, H. Yang, J. Shen, S. Darvishmanesh, P. Luis, A. Sotto, B. Van der Bruggen *J. Membr. Sci.* **2015**, *477*, 183-193.

[6] J. Lin, W. Ye, M.-C. Baltaru, Y. P. Tang, N. J. Bernstein, P. Gao, S. Balta, M. Vlad, A. Volodin, A. Sotto *J. Membr. Sci.* **2016**, *514*, 217-228.
